# A case report of mitochondrial myopathy with membranous nephropathy

**DOI:** 10.1186/s12882-022-02710-0

**Published:** 2022-03-04

**Authors:** Minchao Cai, Qing Yu, Jinfang Bao

**Affiliations:** Department of Nephrology, Shanghai General Hospital, Shanghai Jiao Tong University School of Medicine, No. 100, Haining Road, Shanghai, 200080 People’s Republic of China

**Keywords:** Mitochondrial myopathy, Membranous nephropathy, mtDNA mutation

## Abstract

**Background:**

MtDNA 3243 A > G mutation leads to mitochondrial myopathies with predominant hyperlactatemia. Given the ubiquitous nature of mitochondria, cellular dysfunction can also appear in tissues with high metabolic turnover; thus, there can be cardiac, digestive, ophthalmologic, and kidney complications. MtDNA 3243 A > G mutation has been shown to be with renal involvement in the previous cases of which are FSGS and tubularinterstitial nephritis.

**Case presentation:**

We report a case of patient who had the mitochondrial myopathy with mitochondrial DNA (mtDNA) 3243 A > G mutation diagnosed membranous nephropathy by kidney biopsy, which was never reported before. Our patient was found to have chest tightness and shortness of breath with hyperlactatemia and was diagnosed mitochondrial myopathy with mtDNA 3243 A > G mutation 11 months ago. Acute kidney injury occurred with hyperuricemia (urid acid 1011umol/L) which may be associated with mtDNA mutation. Since then, persistent proteinuria was also found and the 24-h urine protein quantitative was around 2 g. Kidney biopsy was performed and the result was consistent with membranous nephropathy, with abnormal mitochondria seen in renal tubules by electron microscopy.

**Conclusions:**

Patients with mitochondrial myopathy could also have renal presentation of membranous nephropathy. Patients with mtDNA mutation may have various renal manifestations so that more attention should be paid on their kidneys.

## Background

Mitochondrial cytopathies are rare inherited diseases with multisystem manifestations [1]. A adenine to guanine substitution at nucleotide 3243 of the mitochondrial DNA (mtDNA; m.3243 A > G) which affects the tRNA^Leu(UUR)^ gene, has been shown to cause multiple clinical manifestations such as mitochondrial myopathy, encephalopathy, lactic acidosis and stoke-like episodes [2, 3]. The kidneys, as organs with high energy requirements, are rich in mitochondria and can be affected in mitochondrial cytopathies [4]. We describe a case of chronic kidney disease with membranous nephropathy in a patient of mitochondrial myopathy with the mtDNA 3243 A > G mutation.

## Case presentation

A 42-year-old man of mitochondrial myopathy with the mtDNA 3243 A > G mutation presented with persistent proteinuria. Ten months ago, the patient was taken to the emergency room (ER) due to chest tightness and shortness of breath. The patient was conscious with the blood pressure of 150/60 mmHg. Blood gas analysis showed hyperlactatemia, with the maximum of 24 mmol/L. At the same time, dysfunction also presented in the heart, liver and kidneys. The patient was then admitted to the ICU. During ICU hospitalization, tracheal intubation and tracheotomy were performed as aerobic saturation decreased. Blood purification treatment was performed to reduce lactic acid. The patient had the history of poor tolerance to activities since childhood. The patient’s mitochondrial gene test indicated ‘MT-TL1m.3243A > G’ mutation. Muscle biopsy was also performed. The structure of myofibril was destroyed with the disordered arrangement. The mitochondrial cristae were disordered with increased volume. And the internal structure of mitochondria was incomplete. In some mitochondria, crystalline inclusions were arranged in a ‘parking lot’ pattern, and lipid droplets were deposited. Therefore the patient was diagnosed of mitochondrial myopathy with the mtDNA 3243 A > G mutation.

**Fig. 1:**
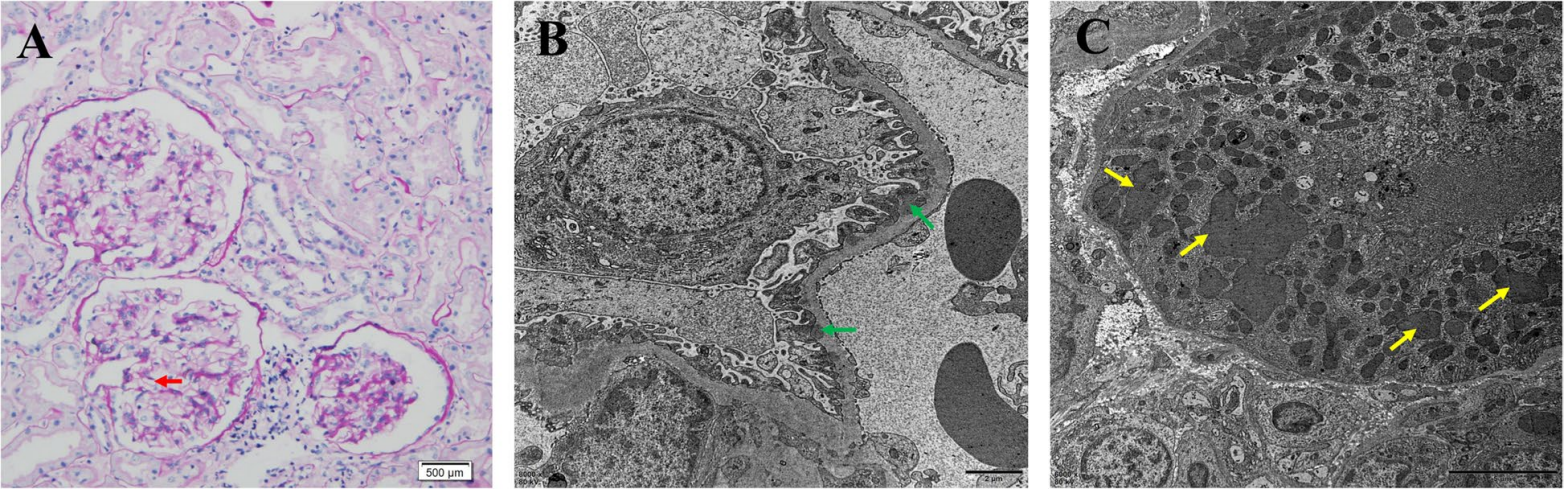
Kidney biopsy findings. **A** Light microscopy shows that the glomerular basement membrane was stiff and thickened (red arrow). **B** Electron microscopy shows subepithelial electron-dense deposits (green arrows) with podocyte foot-process effacement. **C** Few malformed mitochondria were in the renal tubules by electron microscopy (yellow arrows)

Uric acid increased abnormally to 1011umol/L when the patient was in ER, while serum creatinine was 70umol/L. Then the serum creatinine increased up to 172.2umol/L within 48 h, which can be diagnosed acute kidney injury (AKI). The serum creatinine returned to normal soon after the treatment of fluid infusion and blood purification. Proteinuria was also observed at that time and continued till now. The 24-h urine protein quantitative was around 2 g. Kidney biopsy was done in July 2021. It revealed, on light microscopy, 20 glomeruli, 2 being globally sclerotic. There was no cell proliferation or inflammatory cell infiltration in the rest of the glomeruli. The glomerular basement membrane was stiff and thickened. And the capillary loops were open. Renal tubular epithelial cells were swelling (Fig. 1A). On immunofluorescence, there was granular mesangial staining for immunoglobulin G (IgG) (2 +) and C3 (1 +). There was no glomerular positivity for IgA, IgM and C1q. Electron microscopy reveled subepithelial electron-dense deposits with podocyte foot-process effacement (Fig. 1B). Obvious gathering of mitochondria in podocytes was not observed while few malformed mitochondria were seen in the renal tubules (Fig. 1C). Steroid was not given to the patient. The patient was recommended to undergo treatment with vitamin C, vitamin E and idebenone. Recently the proteinuria remained at about 2g.

## Discussion and Conclusions

This case report identifies the genetic mutation at mtDNA 3243 with membranous nephropathy (MN). The mtDNA 3243 A > G mutation in the tRNALeu(UUR) gene is one of the most common mtDNA point mutations. This mutation was initially described in patients with mitochondrial myopathy, encephalopathy, lactic acidosis and stoke-like episodes (MELAS) syndrome [5, 6]. The phenotypic expression of the mtDNA 3243 A > G mutation can be highly variable and causes a wide range of clinical manifestations, including muscle weakness, exercise intolerance, failure to thrive, developmental delay, progressive encephalopathy, migraine, peripheral neuropathy and visual complaints due to ophthalmoplegia [5, 7]. Our patient had long-term exercise intolerance and muscle weakness. The main manifestation of this patient in ICU hospitalization was obvious hyperlactatemia. The gene detection was performed and the results showed that it was mtDNA 3243 A > G mutation. The muscle biopsy further confirmed the diagnosis of mitochondrial myopathy.

Mitochondrial dysfunction can affect multiple tissues, especially tissues with high energy demands. Proximal tubule cells have high metabolic rates and are rich in mitochondria, but lack the capability to synthetize ATP anaerobically from glycolysis [8]. Thus kidneys are vulnerable to mitochondrial dysfunction. Renal involvement was reported in some patients with mtDNA 3243 A > G mutation [7, 9]. The patient was found to have obviously elevated uric acid, more than 1000umol/L, while the serum creatinine was normal when he was taken to the ER. After the patient’s condition was controlled, the patient’s uric acid was still around 500umol/L.It was reported that in single cases renal involvement of mitochondrial disorders was also manifested of hyperuricemia [10–12]. We speculated that mitochondrial myopathy may be the cause of the increased uric acid in this case. Massive uric acid further blocked renal tubules and resulted in AKI, with another ‘accomplice’, hyperlactatemia.

From the histological standpoint, most patients had FSGS lesions, but few cases of tubulointerstitial nephritis have also been described [7, 9]. And it was reported that in patients with mitochondrial myopathy, only a few patients had abnormal mitochondrial accumulation in podocytes or renal tubular epithelial cells, while no similar changes were observed in most patients [4, 9, 13]. Continuous proteinuria was found in our patient since ICU hospitalization and the 24-h urinary protein quantity was around 2 g per day. The renal injury of our patient was consistent with MN. No obvious mitochondrial aggregation was found in podocytes, while a small number of malformed mitochondria in the renal tubules were found. In the previous cases, MN was not reported in patients with mitochondrial myopathies. However, mitochondrial aggregation was not observed in podocytes in our patient. It may be the same as mitochondrial abnormalities are rarely observed in the mitochondrial myopathy patient with FSGS. Few malformed mitochondria in the renal tubules might be the evidence that renal involvement in our patient. At present, our patient is stable for mitochondrial myopathy with taking vitamins and idebenone. No additional treatment was given to our patient after he was diagnosed of MN by kidney biopsy. As the symptoms of mitochondrial myopathy stabilized, proteinuria gradually decreased. It is speculated that MN in this patient may be secondary to mitochondrial myopathy. It is also possible that we observed in our patient the chance co-occurrence of 2 unrelated disorders. Nonetheless, our findings suggest that the manifestations of kidney in mitochondrial myopathy may not only be FSGS and tubulointerstitial nephritis. Renal biopsy should be performed in patients with mitochondrial myopathies who show renal injury.

In conclusion, we reported a case with MN in the genetic mutation at mtDNA 3243. It was suggested that, addition to FSGS, patients harboring mutations in the tRNALeu(UUR) could also manifest MN in kidney. It is possible that kidney disease in mitochondrial myopathies may be subclinical or overshadowed by extrarenal manifestations and thus potentially under-reported. Patients with mtDNA mutation should be had more attention on their kidneys.

## Data Availability

The data used for this case report is available upon reasonable request.

## Abbreviations

mtDNA: Mitochondrial DNA
ER: Emergency Room
AKI: Acute Kidney Injury
MN: Membranous Nephropathy
